# Towards a measurement of internalization of collaboration scripts in the medical context – results of a pilot study

**DOI:** 10.3205/zma000974

**Published:** 2015-08-17

**Authors:** Jan Kiesewetter, Martin Gluza, Matthias Holzer, Barbara Saravo, Laura Hammitzsch, Martin R. Fischer

**Affiliations:** 1 Klinikum der Universität München, Institut für Didaktik und Ausbildungsforschung in der Medizin, München, Deutschland; 2 Ludwig-Maximilians-Universität München, Munich Center of the Learning Science (MCLS), München, Deutschland

**Keywords:** collaboration, teamwork, collaboration scripts, internalization

## Abstract

**Background: **Collaboration as a key qualification in medical education and everyday routine in clinical care can substantially contribute to improving patient safety. Internal collaboration scripts are conceptualized as organized - yet adaptive – knowledge that can be used in specific situations in professional everyday life. This study examines the level of internalization of collaboration scripts in medicine. Internalization is understood as fast retrieval of script information.

**Goal: **The goals of the current study were the assessment of collaborative information, which is part of collaboration scripts, and the development of a methodology for measuring the level of internalization of collaboration scripts in medicine.

**Method:** For the contrastive comparison of internal collaboration scripts, 20 collaborative novices (medical students in their final year) and 20 collaborative experts (physicians with specialist degrees in internal medicine or anesthesiology) were included in the study. Eight typical medical collaborative situations as shown on a photo or video were presented to the participants for five seconds each. Afterwards, the participants were asked to describe what they saw on the photo or video. Based on the answers, the amount of information belonging to a collaboration script (script-information) was determined and the time each participant needed for answering was measured. In order to measure the level of internalization, script-information per recall time was calculated.

**Results:** As expected, collaborative experts stated significantly more script-information than collaborative novices. As well, collaborative experts showed a significantly higher level of internalization.

**Conclusions:** Based on the findings of this research, we conclude that our instrument can discriminate between collaboration novices and experts. It therefore can be used to analyze measures to foster subject-specific competency in medical education.

## Zusammenfassung

**Hintergrund: **Kooperation als eine Schlüsselqualifikation in der medizinischen Ausbildung und im medizinischen Alltag kann einen entscheidenden Beitrag zur Verbesserung der Patientensicherheit leisten. Unter internationalen Kooperationsskripts wird Wissen verstanden, welches zugleich in organisierter und adaptiver Form vorliegt. Sie können situationsspezifisch im Berufsalltag abgerufen und eingesetzt werden. Die vorliegende Studie untersucht die Internalisierung von Kooperationsskripts in der Medizin. Internalisierung wird als schnelle Abrufbarkeit von Skriptinformationen verstanden.

**Ziel: **Das Ziel der Untersuchung war die Erfassung von Informationen, die Bestandteil von Kooperationsskripts sind, sowie die Entwicklung einer Methode zur Messung des Internalisierungsgrades von Kooperationsskripts in der Medizin.

**Methode:** Für den kontrastiven Vergleich der internationalen Kooperationsskripts wurden insgesamt 20 Kooperationsnovizen (Studierende im Praktischen Jahr (PJ)) und 20 Kooperationsexperten (Fachärzte der Inneren Medizin oder Anästhesiologie) in die Studie einbezogen. Den Probanden wurden jeweils für fünf Sekunden acht kooperationstypische Situationen in Form eines Fotos oder eines Videos präsentiert. Anschließend wurde zu jeder Situation gefragt, was auf dem Bild zu sehen war. Aus den Antworten wurde die Anzahl von Informationen, die zu einem Kooperationsskript (Skriptinformationen) gehören, nach einem etablierten, theoriebasierten Kodierschema erfasst sowie die Zeit, die ein Proband zum Antworten benötigte, gemessen. Als Maß für den Internalisierungsgrad wurden die Skriptinformationen pro Zeiteinheit berechnet.

**Ergebnisse: **Verglichen mit Kooperationsnovizen nannten Kooperationsexperten signifikant mehr Skriptinformationen. Kooperationsexperten zeigten zudem einen signifikant höheren Internalisierungsgrad.

**Schlussfolgerung:** Die gewonnenen Erkenntnisse belegen, dass das entwickelte Messinstrument zwischen Novizen und Experten im Bereich Kooperation diskriminieren kann. Es kann somit eingesetzt werden, um Maßnahmen zur fachspezifischen Vermittlung von Kooperation in der medizinischen Lehre zu untersuchen und zu bewerten.

## Background

**Figure 1:**
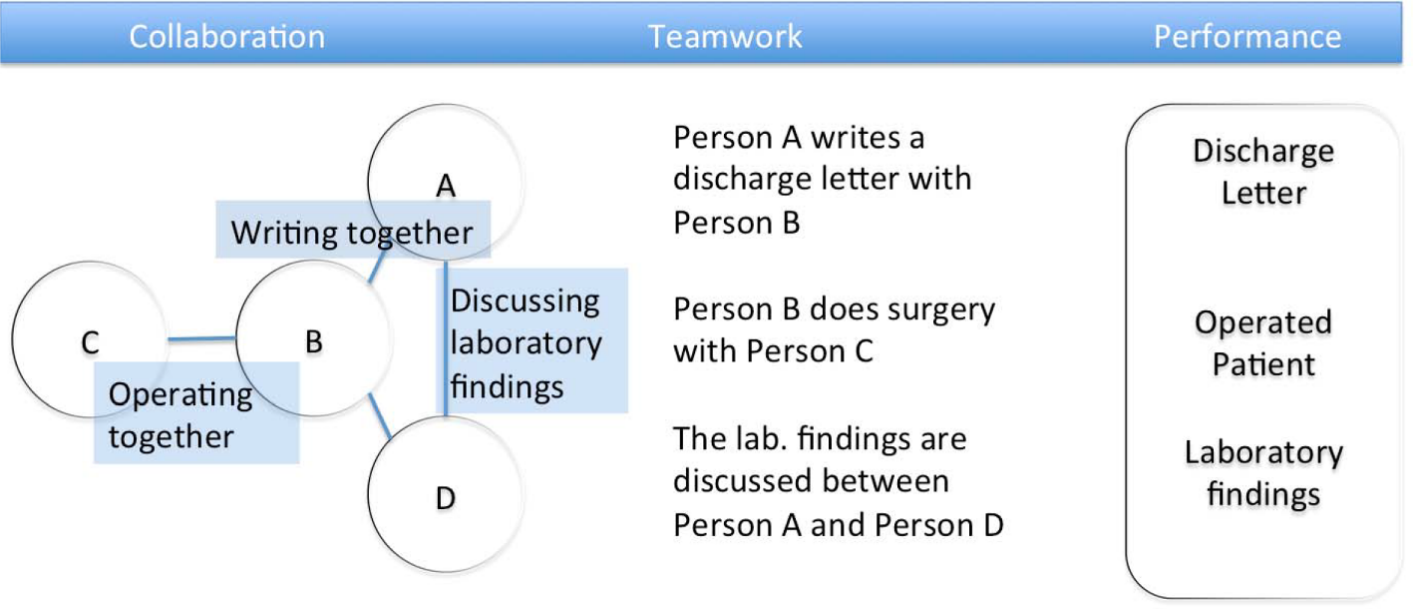
The relationship between teamwork, collaboration and performance.

The ability to collaborate in the health professions has been identified and called for as one of the core competencies for the 21^st^ century by the World Health Organization [1]. Collaboration in this sense means the process that enables teamwork [2] (see Figure 1 (Fig. 1)). When clinical teamwork in the hospital is efficient, it has been associated with less errors [3], [4] and greater work satisfaction [5]. Within the development of the national competency-based learning catalogues of medicine and dental medicine (Nationaler Kompetenzbasierter Lernzielkatalog Medizin (NKLM) und Zahnmedizin (NKLZ)), it has been defined as a goal to integrate teamwork competencies through the role of a physician as member of a team into medical curricula in Germany [http://www.mft-online.de/files/2012_omft_hickel_fischer.pdf, last viewed June 25^th^, 2015]. An improvement of teamwork through the consideration of collaboration within trainings is desirable but not easy to realize [6]. 

Domains outside medicine found that knowledge regarding collaboration is associated with performance and the learning in collaborative situations [7]. The internal representation of the sequence of actions has been conceptualized as *internal* *collaboration script* [8], including knowledge, which is organized and flexible with describable sequences of activities in a given context [9]. In this paper, the described context is medical teams.

The conceptual supplement to internal collaboration scripts are external collaboration scripts, which include information from the outside to structure collaborative processes. Script theory indicates that such external information is internalized over time and develops the internal collaboration script for this situation. Through this internalization, external collaboration scripts are not necessary anymore. 

With an example from the medical context the development of an internal collaboration script will now be illustrated: rotations through different medical subdisciplines are an integral part of medical education. Students in the final (so-called practical) year in Germany rotate on up to four different internal medicine wards with different core areas within three months. An imaginary student in the beginning of his work on a ward does not know the typical course of events and treatments are not yet known. It is difficult to keep up with the daily routine and be a part of the clinical team. Gradually the student develops adequate collaboration scripts; first through observation and then, through continuous practice, he is able to support the daily routine. The student learns the typical situations and can integrate himself in the daily routine of the ward.

When changing wards, some things change for the student. Besides the personnel, the diseases treated on the wards, the according diagnostic procedures and therapies and, consequently, much of the daily routine. Accordingly, two internal medicine wards with different core areas (i.e. cardiology and nephrology) can differ in the attributes mentioned above. The script developed in one ward is valid only partly and has to be adapted to the new context, or rather new aspects have to be added to the existing script. 

So the collaboration script is supplemented with the new components for the new ward. In the example, the medical student has developed a set of expectations, notions and a repertoire of possible actions from which he can choose the most appropriate for a given situation from two wards [10]. 

When changing wards again, the adequate behavior in changing situations will continue to be easier through continual development and/or adaption of collaboration scripts. The situations are familiar and he has an increased number of available courses of actions compared to the beginning of his work. 

This example points out the theoretical foundation of collaboration scripts. However, little is known regarding the assessment and development of internal collaboration scripts in the context of medicine. To realize goal-oriented trainings in medical education, reliably assessing the existing collaboration scripts of training participants is of great importance. The assessment is seen as challenging because, in addition to knowledge regarding collaboration, knowledge in another domain, the so called content domain, which the people collaborate in, is necessary [11]. Differences in measurement cannot only be traced back to differences in the internal collaboration scripts but also to differences in the specialized knowledge of the content domain. An approach in which the specialized knowledge has not been integrated in the assessment of collaboration scripts has been presented by Kiesewetter, Fischer and Fischer [12]. Controlling the collaboration situation, collaborative expertise has been found to be a rather domain-specific ability. This means that collaboration is related to one content domain and cannot be transferred to another content domain easily. Based on this study, the question is how collaboration scripts of experts and novices can be retrieved in a new content domain. In other domains outside of medicine it was found that collaboration scripts develop through repeated experience and are internalized gradually [13], [14]. The principle of internalization describes that the time of retrieval of internalized components of the script is an indicator of how well the components are linked to one another: The more often components of a script are utilized together the faster they should be retrievable [14]. The measured time for the retrieval of all of the collaboration script information is used as a surrogate parameter for internalization.

A simple example is used as an illustration for the link of the terms “hyperthyroidism” and “cardiac arythmia”. In a task in which the phrase is tested for correctness, the sentence “hyperthyroidism leads to TSH-decrease” verified faster than the sentence “hyperthyroidism leads to cardiac arythmia” and this, in turn, is verified faster than “hyperthyroidism has influence on the metabolism”. Because the terms “hyperthyroidism” and “TSH-decrease” are used immediately together more often, the correctness of the sentence can be verified faster. 

Especially in medicine, the fast retrieval of collaborative actions is of particular importance. To measure the availability of script information, valid measurement instruments are necessary. The goal of the following investigation was the assessment of information, integrated in collaboration scripts as well as the development of a measurement method to quantify the level of internalization.

It is assumed that, based on a better organization and linkage of their internal collaboration scripts, experts possess more elaborate script information and can retrieve it faster than novices. This assumption was used to conceptualize a study in which a group of experts and novices was confronted with a collaborative situation and to investigate whether these groups can be distinguished regarding the amount of script information and the retrieval time. 

## Method

### Participants

Novices (*N*=20, 13 female, 25.8 years in average, *SD*=4.7) and collaborative experts (*N*=20, 8 female, *M*=41.6 years in average, *SD*=7.9) volunteered to participate in the study. The number of participants was analyzed a priori using G*Power (http://www.gpower.hhu.de/) and is large enough to uncover medium-sized effects. All novices were medical students (predominantly in their 5^th^ year) of the Ludwig-Maximilians-University Munich. As collaborative experts, specialist doctors (on average, *M*=15.2 years of professional experience, *SD*=7.7) were recruited from the collaboration-intensive specialties internal medicine and anesthesiology. Through their completed specialty training, it could be verified that the number of collaborative situations should be sufficient and numerous, and that experts should be able to outperform the novices. Further demographic data as well as number of collaboratively worked hours per day were acquired. Physicians were included in the study only when the number of collaboratively worked hours per day exceeded four hours per weekday. The restriction on two specialties and the control of the number of collaboratively worked hours was set to counteract confounding effects of the content and collaboration domain. 

#### Procedure

To enable the utilization of internal collaboration scripts all participants were successively confronted with eight stimuli of four 5-second videos and four photos, each for five seconds. 

**Figure 2:**
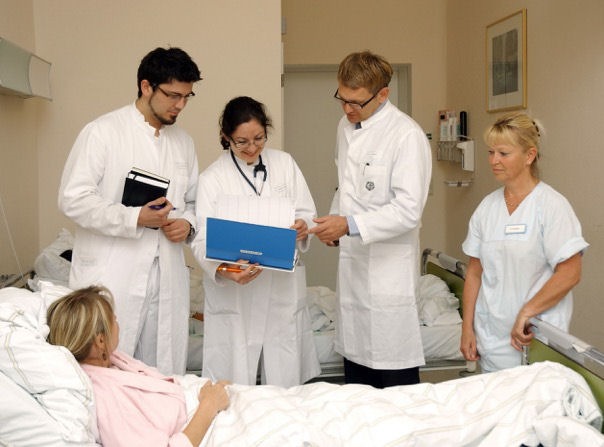
Example of a stimulus of a collaborative situation.

The stimuli showed collaborative situations in the medical context (i.e. an enacted ward round situation). Figure 2 (Fig. 2) is an example of a stimulus of such a collaborative situation. After each of four stimuli, a memory question was asked, while after each of the other four stimuli three script questions were asked. The script questions were asked to simplify the task for novices because they were asking for courses of action more directly. The memory question was: “Please state what you can remember from the just viewed stimulus”. The script questions were

1. “What are the persons doing in the picture/video?”
2. “How does this type of situation typically come to happen?“
3. “What is the most likely way how this situation could continue?“.

It was ensured that the type of stimulus (video/photo) and the type of question (memory/script question) were counterbalanced so that each participant had the combination video-script question at least twice and the combination photo-script question twice. The presentation of the stimuli and the answer of the questions was performed at a laptop computer, which made measuring the time per stimulus for each participant possible via logfiles. Using the logfiles, it was possible to trace back when a participant had sent an answer. The participants were given as much time as they needed to answer the questions.

#### Coding scheme

To analyze the data, a proved and tested coding scheme was used [12], which is focused on collaboration scripts but not content knowledge. The coding scheme included two major categories: superficial and script information. Superficial information included the type of clothing, the color of hair (etc.), which didn’t give any valuable clues regarding the role of the person in the situation. Script information specified activities, sequences of activities, and roles for persons visible in the scene. The coding scheme for script information goes back to the theory-based components of collaboration scripts by Kollar, Fischer & Hesse [8]. Information was coded regarding keywords. If a predefined keyword occurred in one of the answers of a participant, it was either coded as a superficial or a script information. Script information was further subdivided into goals (i.e. use of the word “ward round”), part activities (denomination of specific activities like “retrieve laboratory results”), order (correct denomination of courses of action), role assignment (denomination of persons as “nurse” or “physician”), attributes (denomination of adjectives for persons with regard to teamwork like “dominant”), and typical objects (denomination of typical hospital objects like “patient’s file”).

Answers of participants were first coded and counted by one coder with the use of Microsoft Excel 2010. Afterwards, 10% of the answers where coded by a second, independent person. The inter-rater reliability was Cohens *k*=.84.

#### Data analysis

As a measurement of the level of internalization of a collaboration script, a quotient of the time per answer in seconds and number of script information was calculated.

The difference in the level of internalization between experts and novices was tested using a t-test. Alpha-error was set to .05. If data was used more than once in an analysis, the alpha error level was Bonferroni-corrected. Further, a group comparison between experts and novices regarding the denominated script information was analyzed using ANOVA. Data was analyzed using SPSS 20.0 (IBM Inc.). Specification of effect sizes relate to Cohen *d* [15]. 

## Results

The statistical analysis revealed that collaboration experts (*M_Experts_*=71,65, *SD*=33,23) stated significantly more script information than novices (*M_Novices_*=54,25, *SD*=15,01, *F*(1;38)=4.55; *p*<0,05; *d*=0,69). The difference of means indicates a medium sized effect [16]. The number of script information are illustrated in Table 1 (Tab. 1).

**Figure 3:**
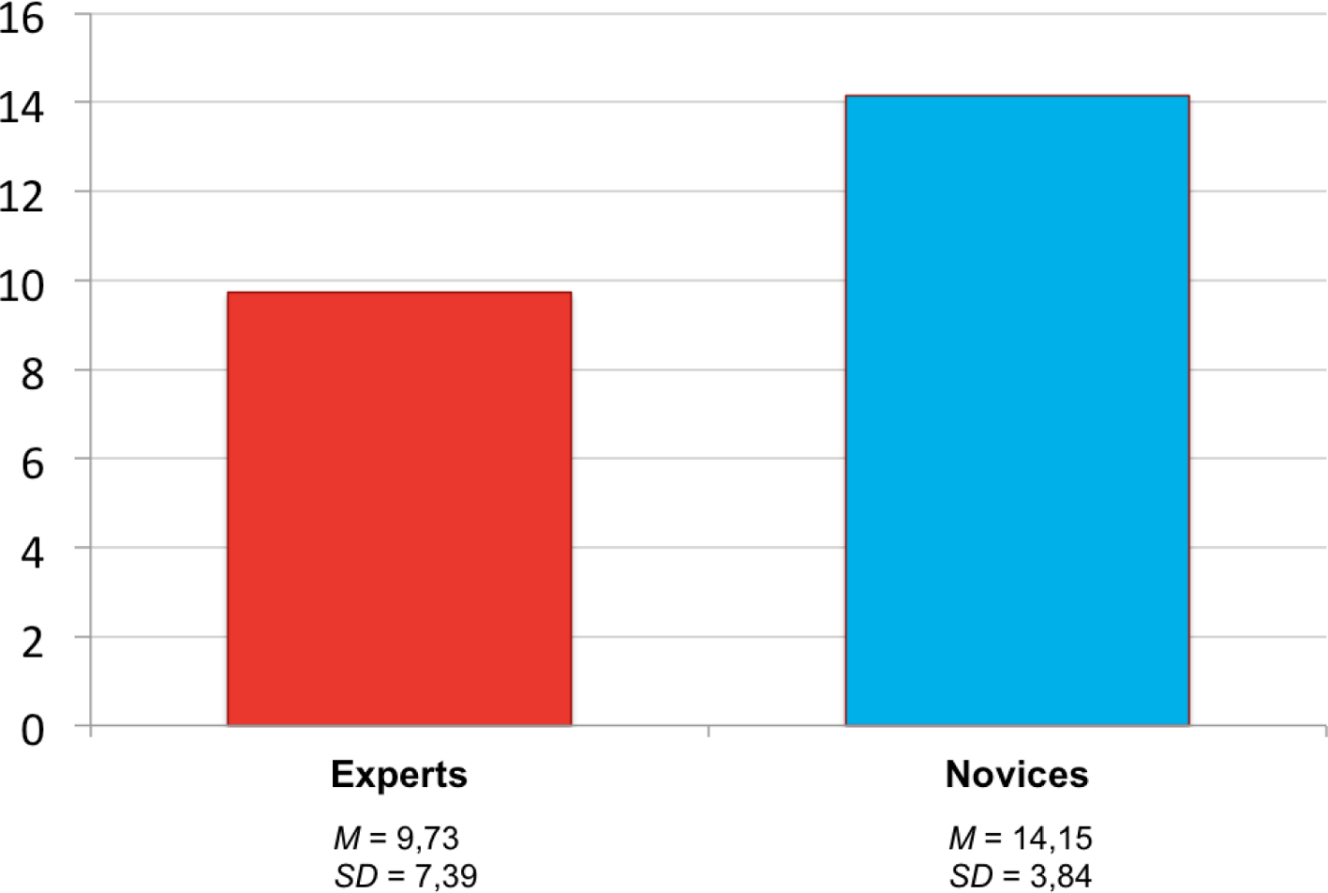
Difference in the level of internalization between experts and novices (means).

As hypothesized, experts had a deeper level of internalization regarding the time (seconds) per script information (*M_Experts_*=9,73, *SD*=7,39) than novices(*M_Novices_*=14,15, *SD*=3,84). From the lower mean of the experts, one can infer that they needed a shorter amount of time to retrieve script information than novices or that more information was retrieved in the same amount of time, both indicating a deeper level of internalization of collaboration scripts of experts. The t-test to analyze the difference in level of internalization between experts and novices is significant with a medium to large effect size (*t*(38)=2,38; *d*=0,75). The comparison is depicted in Figure 3 (Fig. 3). 

## Discussion

This study was designed to test how to quantify the level of script internalization by utilizing the script information per time as surrogate parameter of internalization for the first time. Collaboration experts (physicians from internal medicine and anaesthesiology) differ significantly from novices (medical students) regarding their knowledge about collaborative situations. Methodologically, a presentation of different stimuli to simulate standardized collaborative situations was applied. The study showed that experts could retrieve more internalized script information than novices. Further, the defined level of internalization of collaboration scripts of the experts differed significantly from novices. Experts were not only able to state more script information but also to retrieve them significantly faster. This strengthens the notion that components of a collaboration script can be retrieved faster, the more often they are used together (cf. [14]). The results indicate that during the development of expertise, internal collaboration scripts are extended and stored differently. 

Collaboration as a core qualification is going to be an integral part of medical curricula in Germany and throughout the world [17]. To measure curricular implementations and to support the development of collaborative abilities, internal collaboration scripts and their level of internalization need to be assessed validly. Promising approaches exist for the domain of computer-supported collaborative learning [18]. Ways to advance the present study comprise the use of interventions to validate the measurement approach in the scope of a randomized controlled trial. As a next step, concrete collaborative behavior could be investigated in existing as well as new collaborative situations. This operationalization of internal collaboration scripts in a wider context of application is expected to advance validity to a notable extent.

The measurement instrument presented here is a beneficial means to study trainings of collaboration expertise, demonstrating that collaboration experts have a more efficient comprehension of collaborative situations and can retrieve more possible courses of action. Further, the – assumed – capability of the instrument to show that novices profit significantly from a repeated training of (authentic) collaborative situations should be expressed in two ways: a) the acquired knowledge regarding collaboration should be internalized more deeply and b) the retrieval should be faster.

In addition to the assessment of internal collaboration scripts the question of how learning processes can be supported and scaffolded through external collaboration scripts should be examined. In the literature there are isolated approaches, such as how feedback processes can be advanced using external scripts [19]. Through goal-oriented scaffolding, team members could develop shared knowledge (meaning shared collaboration scripts and transactive memory systems [18]), which could positively influence teamwork on a daily basis. This paper demonstrated a methodical proposal as to how such a learning process can be quantitatively conducted and assessed. Further research in this field could contribute to the implementation of the “collaborate practice-ready health workforce” stipulated by the World Health Organization [1].

### Limitations

The generalizability of the conclusions is limited by the non-interventional study design. Applying an experimental approach, the effects of this study should be tested in order to target more generalizable effects. Another limitation is the large variance in the group of experts regarding the amount of script information and the amount of time. This is possibly due to the assumption that during the time-on-task, other cognitive processes – not assessed in this study – take place, such as the change of attention and motivation. As the collaborative work times were controlled with a questionnaire and the study sample was carefully selected, another explanation for the variance can be that collaboration expertise is determined multifactorially and expressing quite differently. Further studies are necessary to probe these assumptions. Medium sized effects, distinguishing novices from experts do, however, speak for the presented approach.

A possible confound between the content and collaboration domain cannot be eliminated with full certainty. Two measures were taken to counteract this influence. First, the comparison was limited to the disciplines anesthesiology and internal medicine to limit the heterogeneity of content domains. Second, the coding scheme was designed for collaboration scripts and did not measure content knowledge.

## Outlook

In this pilot study, a method to assess the amount of information, integrated in collaboration scripts was applied to test the level of internalization of collaboration scripts in medicine.

Collaboration experts, physicians from internal medicine and anesthesiology stated significantly more collaboration script information per timeframe when compared with novices (medical students). The collaboration experts showed a significant deeper level of internalization. The measurement instrument has the potential to evaluate trainings to teach collaborative competencies in medical education. Other studies to validate the measurement instrument are definitely necessary. 

## Acknowledgements

The first author is grateful for the financial support of the German Academic Exchange Agency during the development of this research.

## Competing interests

The authors declare that they have no competing interests.

## Figures and Tables

**Table 1:**
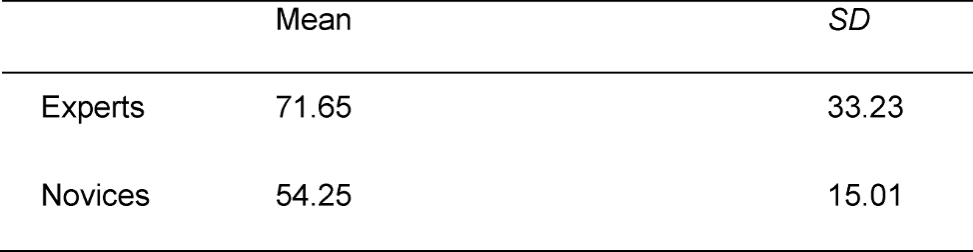
Number of script information of collaboration experts and novices
